# Metformin use mitigates the adverse prognostic effect of diabetes mellitus in chronic obstructive pulmonary disease

**DOI:** 10.1186/s12931-019-1035-9

**Published:** 2019-04-05

**Authors:** Te-Wei Ho, Chun-Ta Huang, Yi-Ju Tsai, Angela Shin-Yu Lien, Feipei Lai, Chong-Jen Yu

**Affiliations:** 1 Graduate Institute of Biomedical Electronics and Bioinformatics, College of Electrical Engineering and Computer Science, National Taiwan University, Taipei, Taiwan; 2 Department of Surgery, College of Medicine, National Taiwan University, Taipei, Taiwan; 3 Department of Internal Medicine, National Taiwan University Hospital, No. 7, Chung-Shan South Road, Taipei, 100 Taiwan; 4 Graduate Institute of Clinical Medicine, National Taiwan University, Taipei, Taiwan; 5 Graduate Institute of Biomedical and Pharmaceutical Science, College of Medicine, Fu Jen Catholic University, New Taipei City, Taiwan; 6 School of Nursing, College of Medicine, Chang Gung University, Taoyuan City, Taiwan; 7 Division of Endocrinology and Metabolism, Department of Internal Medicine, Chang Gung Memorial Hospital Linkou Branch, Taoyuan City, Taiwan

**Keywords:** Chronic obstructive pulmonary disease, Comorbidity, Diabetes mellitus, Metformin, Outcome

## Abstract

**Background and objective:**

Among patients with chronic obstructive pulmonary disease (COPD), diabetes mellitus (DM) is a common comorbidity and is probably associated with increased systemic inflammation and worse prognosis. Metformin, with its pleiotropic anti-inflammatory and antioxidant actions, may offer theoretical benefits in COPD patients with DM. Thus, this study aimed to investigate the effects of DM and metformin use on mortality in the clinical trajectory of COPD.

**Methods:**

This was a retrospective cohort study comprising patients with spirometry-confirmed COPD and an age of ≥40 years from 2008 to 2014. The primary outcome of interest was all-cause mortality. We evaluated the effects of DM on mortality through the clinical course of COPD and we also assessed the impact of metformin use on survival of the COPD population.

**Results:**

Among 4231 COPD patients, 556 (13%) had DM, and these patients had 1.62 times higher hazards of 2-year mortality than those without DM (95% confidence interval [CI], 1.15–2.28) after adjusting for age, gender, COPD stage, comorbidities and prior COPD hospitalization. Over a 2-year period, metformin users had a significantly lower risk of death (hazard ratio, 0.46; 95% CI, 0.23–0.92) compared with non-metformin users in patients with coexistent COPD and DM. Moreover, metformin users had similar survival to COPD patients without DM.

**Conclusions:**

This study shows that DM is associated with an increased risk of death in COPD patients and metformin use seems to mitigate the hazard. Our findings suggest a potential role of metformin in the management of DM in COPD.

**Electronic supplementary material:**

The online version of this article (10.1186/s12931-019-1035-9) contains supplementary material, which is available to authorized users.

## Introduction

Chronic obstructive pulmonary disease (COPD) is one of the most important global health problems that affects 380 million individuals worldwide [1]. COPD is characterized by persistent airflow limitation that is usually progressive and associated with an increased chronic inflammatory response in airways and lungs to noxious gases and particles [2]. In addition to pulmonary abnormalities, COPD is considered a systemic inflammatory disorder that exerts a variety of effects on extrapulmonary tissues and organs [3]. In the past 2 decades, the approach towards patients with COPD has shifted from nihilism to viewing it as a preventable and treatable disease [2, 4]. Comorbidities contribute to the overall severity in individual patients and also play an important role in the prognosis of COPD [2, 5]. In fact, the presence of almost all comorbidities has increased in the COPD population, particularly in those that are smoking-related, and one-half of the increased healthcare utilization and costs found in COPD patients are attributable to these comorbidities [6].

Diabetes mellitus (DM) is a recognized clinical entity for more than 2 centuries. Over the past 30–40 years, the treatment modalities available for patients with DM and patient outcomes have dramatically improved. Despite medical advances, the global prevalence of DM has continued to increase and will reach an estimated 4.4% by 2030 [7]. DM is a common comorbid condition in patients with COPD and is probably associated with increased systemic inflammation and poor outcomes [5, 8–10]. However, some data suggest a positive effect related to DM in COPD. A recent systematic review found that DM is associated with better short-term survival following COPD exacerbations [11]. The protective effect is mirrored in a cohort study by McGhan et al. who showed that DM is associated with a decreased risk of rehospitalisation after an COPD exacerbation [12]. To date, the protective effect of DM on COPD and its mechanism resulting in such beneficial outcomes remain uncertain. Preferential care of DM patients could represent a contributing factor, or hypothetically, antidiabetic agents used to control hyperglycaemia may modify the clinical course of coexistent COPD and DM.

Metformin, which is a widely available oral antidiabetic medication, is the recommended first-line treatment for type 2 DM and is associated with a reduced risk of cardiovascular events and death [13]. Metformin possesses pleiotropic anti-inflammatory and antioxidant actions and may play a role in patients with COPD and DM besides its glucose-lowering effect [14, 15]. .An animal study has provided evidence that metformin reduces glucose flux across the airway epithelium to limit bacterial growth in the presence of hyperglycaemia [16], a risk factor for increased exacerbation frequency and poor outcomes from COPD exacerbations [17, 18]. Clinical observations also indicated that metformin use is associated with increased inspiratory muscle strength and improvements in dyspnoea, health status, and lung function in patients with both COPD and DM [19, 20]. Thus, metformin may potentially offer theoretical benefits in COPD patients with DM.

In this regard, we designed a retrospective cohort study to assess the effects of DM and metformin use on all-cause mortality in the clinical course of COPD, controlling for common confounders accompanying COPD and DM.

## Methods

### Study design and subjects

This was a retrospective cohort study conducted at the National Taiwan University Hospital (NTUH), a 2600-bed tertiary-care referral centre in Taiwan. The medical records from the Integrated Medical Database, National Taiwan University Hospital (NTUH-iMD) from March 2008 to December 2014 were used in this study. The NTUH-iMD database contains all information, such as demographics, diagnosis, pharmacies, procedures, laboratories, clinical patient notes, nursing notes and death records, of inpatient and outpatient visits to the NTUH since 2006, and is maintained up-to-date by the NTUH. We identified individuals with COPD, defined by the presence of airflow limitation (proportion of the forced vital capacity exhaled in the first second [FEV_1_/FVC] < 0.7) and aged ≥40 years. The index date was defined as the date of receipt of the first diagnosis of COPD. Patients were generally assessed and treated based on the contemporary Global Initiative for Chronic Obstructive Lung Disease (GOLD) guidelines [4, 21]. The study protocol was approved by the Research Ethics Committee of the NTUH (201707080RINA).

### Identification of DM and metformin use

From the index date, a 1-year diabetes ascertainment window was created to classify patients with COPD into those with or without DM as a comorbidity. The diagnosis of incident and prevalent DM was established by either of the following criteria during the diabetes ascertainment period: (a) International Classification of Diseases (ICD) codes for DM plus a prescription of oral antidiabetics or insulin; or (b) ICD codes for DM plus a fasting blood glucose level of ≥126 mg/dl, a random blood glucose level of ≥200 mg/dl or a haemoglobin A1c (HbA1c) value of ≥6.5%. Patients with a diagnosis of type 1 DM were excluded from the study. Metformin use was recorded from the prescriptions filled following the diabetes ascertainment period and was classified as follows: if patients were using metformin within 30 days after the end of the ascertainment period, they were categorized as metformin users over the follow-up period in line with the principle of intention-to-treat analysis. We excluded study subjects with chronic kidney disease (CKD) stage 3b or higher from the metformin analysis because it is a relative contraindication to the use of metformin and may confer a confounding effect [22].

### Data collection

Data regarding the following known risk factors associated with long-term outcomes in COPD and potential confounders were obtained: age, gender, body mass index, hypertension, cerebrovascular disease, heart failure, coronary artery disease, malignancy, CKD, FEV_1_, and the number of hospitalization within 1 year after the index date [23]. Data on HbA1c values and number of antidiabetic classes used were also collected [24]. The CKD stages were classified as suggested by the National Kidney Foundation Kidney Disease Outcomes Quality Initiative and the Kidney Disease: Improving Global Outcomes [25]. The COPD stages were determined using the GOLD criteria in 2013 [21].

**Fig. 1:**
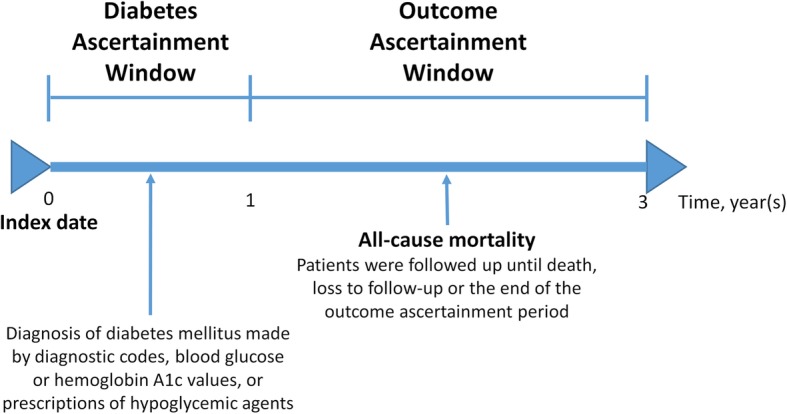
Diabetes and outcome ascertainment periods in the primary analysis

The primary clinical outcome was all-cause mortality, which was evaluated beginning at 1 year after the index date until 3 years after the index date, i.e., the 2-year period beyond the diabetes ascertainment window (Fig. 1). An advantage to this approach was the minimization of the potential for immortal time bias by fixing DM status before the outcome ascertainment period [26]. Patient survival status was determined as the time to death from the end of the diabetes ascertainment window to the date of death, time to the end of the outcome ascertainment window for a maximal total of 2 years for the survivors, or time to the last follow-up date for subjects lost to follow-up before the end of the outcome ascertainment window. Death of the patients was ascertained by death certificates in the NTUH-iMD database. The loss to follow-up was defined as ≥3 months late for a scheduled visit and not yet classified as dead.

### Statistical analysis

Data were primarily analysed using the SPSS software (Version 20.0; IBM Corp., Armonk, NY, US), and the statistical significance was set at *P* < 0.05. The comparisons of characteristics between COPD patients with and without DM were compared using the chi-square or Student’s t-test, as appropriate. Univariate Cox regression analysis with all-cause mortality as the outcome was used to calculate crude hazard ratios (HRs) for DM, age, gender, BMI, GOLD stage, comorbidities of interest and prior COPD hospitalization. Multivariate Cox regression analysis was performed with all-cause mortality as the outcome variable predicted by DM status. All covariates were entered into the final model without model selection. The survival curves were plotted using the Kaplan-Meier method and compared using the log-rank test. The effect of metformin on time to all-cause mortality during the 2-year follow-up period was analysed using survival analysis and Cox regression modelling in a similar manner as described earlier. Schoenfeld residuals was used to test the assumption of proportional hazards for variables in the final model using the Stata software (Version 11; StataCorp, College Station, TX, US), and global test *P* > 0.05 indicated no violation of the assumption. We also used competing risks regression to analyse the effect of metformin on all-cause mortality, treating loss to follow-up as a competing risk via the stcrreg function in Stata.

## Results

### Baseline characteristics of COPD patients stratified by DM status

A total of 5597 patients with spirometry-confirmed COPD were identified between March 2008 and December 2014. Of those, 4231 subjects had at least 1-year follow-up period from the index date of COPD diagnosis to identify accurately the DM status. Baseline demographics and clinical features are displayed in Table 1. The mean age was 72.0 years (standard deviation, 10.4 years), and 83% were men. The male predominance in this study was similar to a recent national epidemiology survey of COPD patients in Taiwan [27]. Of the 4231 patients, 556 (13%) had DM (Additional file 1), and they were older than patients without DM. With regard to comorbid illnesses, DM patients were more likely to have hypertension, cerebrovascular disease, heart failure, coronary artery disease, malignancy, and CKD. No significant differences were observed between the two groups in the distribution of GOLD stages or the number of hospitalization within 1 year after the index date.

**Table 1:** Baseline characteristics of patients with chronic obstructive pulmonary disease, stratified by diabetes mellitus status (*N* = 4231)

Characteristic	Total	DM	Without DM	*P* value^‡^
	*N* = 4231	*N* = 556	*N* = 3675	
Age, years	72.0 ± 10.4	73.4 ± 9.3	71.8 ± 10.6	< 0.001
≥ 65	3188 (75)	451 (81)	2737 (75)	0.001
Male gender	3525 (83)	456 (82)	3069 (84)	0.378
BMI (*N* = 3634; 527; 3107)	23.3 ± 3.9	24.6 ± 4.2	23.1 ± 3.8	< 0.001
< 18.5	372 (10)	32 (6.1)	340 (11)	< 0.001
18.5–24	1760 (48)	217 (41)	1543 (50)	
> 24	1502 (41)	278 (53)	1224 (39)	
GOLD stage				
1	1273 (30)	148 (27)	1125 (31)	0.090
2	1987 (47)	271 (49)	1716 (47)	
3	818 (19)	122 (22)	696 (19)	
4	153 (3.6)	15 (2.7)	138 (3.8)	
Comorbidity				
Hypertension	1578 (37)	343 (62)	1235 (34)	< 0.001
Cerebrovascular disease	328 (7.8)	67 (12)	261 (7.1)	< 0.001
Heart failure	423 (10)	104 (19)	319 (8.7)	< 0.001
Coronary artery disease	801 (19)	183 (33)	618 (17)	< 0.001
Malignancy	581 (14)	97 (17)	484 (13)	0.006
Chronic kidney disease	274 (6.5)	84 (15)	190 (5.2)	< 0.001
Hospitalization, No.^a^	0.04 ± 0.27	0.04 ± 0.22	0.05 ± 0.27	0.752
≥ 1	150 (3.5)	21 (3.8)	129 (3.5)	0.751

*BMI* body mass index, *DM* diabetes mellitus, *GOLD* Global Initiative for Chronic Obstructive Lung Disease

^a^Within 1 year after the index date

^‡^ Comparison between patient groups with and without DM

### Effects of DM on mortality in COPD patients

**Fig. 2:**
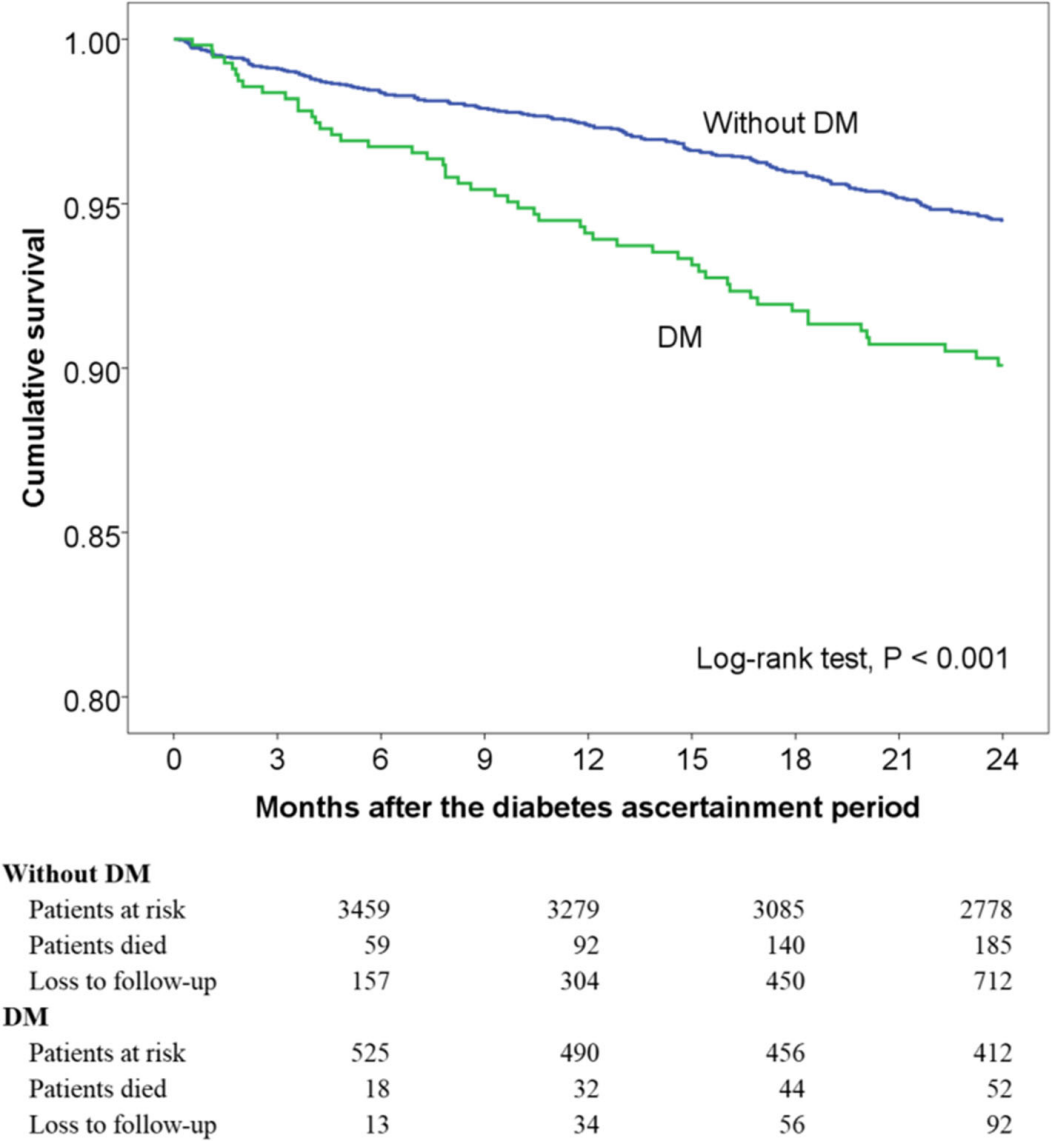
Unadjusted Kaplan–Meier curves for survival in all chronic obstructive pulmonary disease patients with and without diabetes mellitus (*N* = 4231). The mean follow-up period of the patients was 21.3 months, with 90,068 person-months at risk. DM, diabetes mellitus

Within 2 years after the diabetes ascertainment period, COPD patients with DM had a higher probability of mortality than those without DM (*P* < 0.001; Fig. 2). Adjusted HRs for the Cox regression model with mortality in the 2-year follow-up period as the outcome are shown in Additional file 2. After adjusting for age, gender, GOLD stage, comorbidities, and prior COPD hospitalization (global test *P* = 0.305), patients with DM had 1.62 times higher hazards of 2-year mortality than those without DM (95% CI [confidence interval], 1.15–2.28; *P* = 0.006).

### Effects of metformin on mortality among COPD subjects with DM

**Fig. 3:**
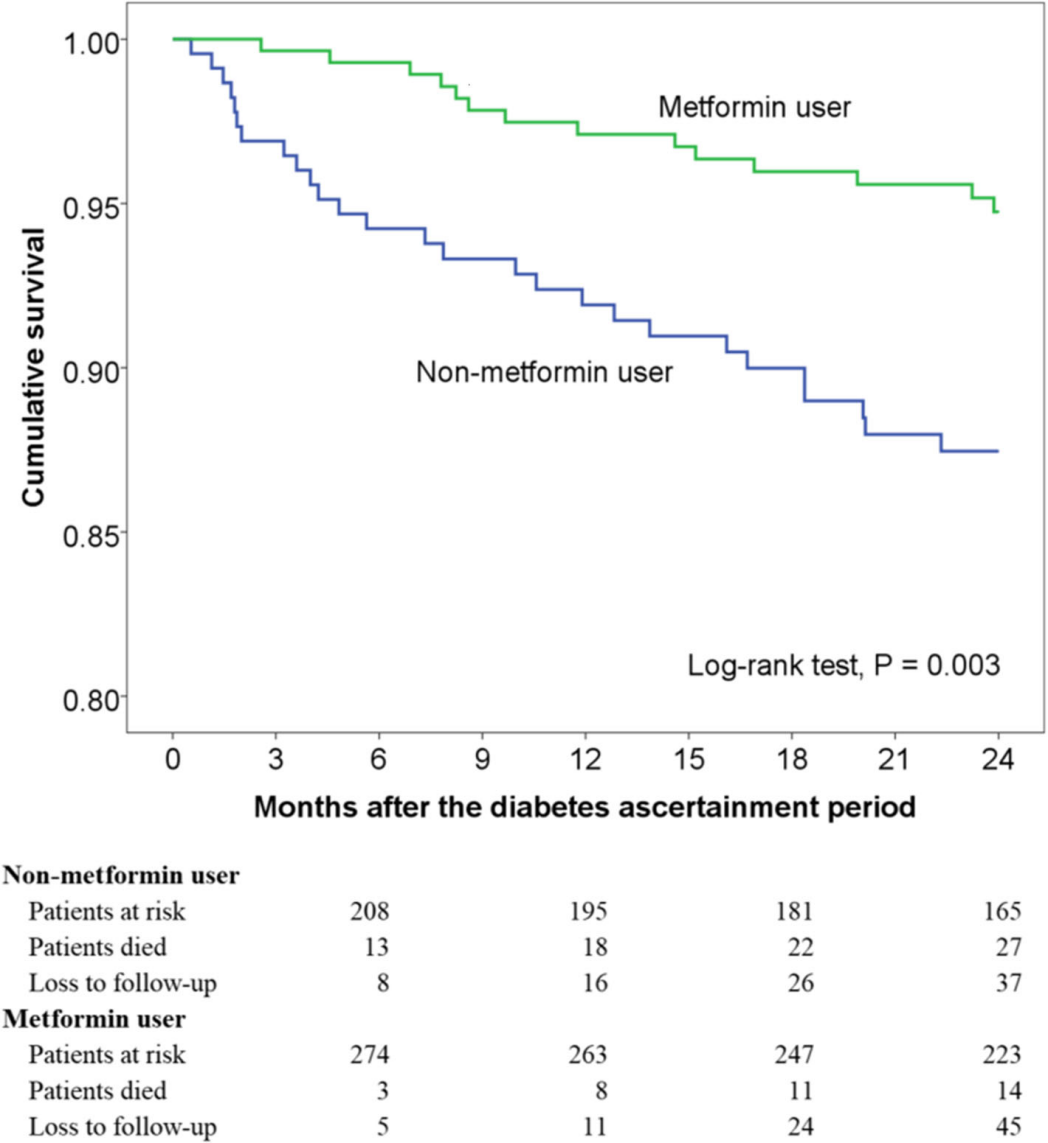
Unadjusted Kaplan-Meier estimates of time to mortality among diabetic patients in chronic obstructive pulmonary disease according to metformin use (*N* = 511). The mean follow-up period of the patients was 21.4 months, with 10,958 person-months at risk. Patients with chronic kidney disease stage 3b or higher were excluded due to concerns for confounding

Of the 556 patients with DM in this study, 45 were excluded because of CKD stage 3b or higher, a relative contraindication to metformin use, to eliminate the potential confounding effect. Of the remaining 511 COPD patients with DM, 282 received metformin, and 229 received other antidiabetic agents (Additional file 3) at the initiation of the 2-year follow-up period. Patients on metformin had significantly lower mean age and proportion of heart failure and malignancy (Table 2). During the 2-year follow-up period, patient survival was significantly better in metformin users by using the log-rank test of Kaplan-Meier survival curves (*P* = 0.003; Fig. 3). In a Cox regression model controlling for age, gender, GOLD stage, comorbidities, prior COPD hospitalization, and number of antidiabetic classes (global test *P* = 0.660), patients in the metformin group had 0.46 hazards of death compared with those in the non-metformin group (95% CI, 0.23–0.92; *P* = 0.028; Additional file 4). When loss to follow-up was accounted for as a competing risk, a lower risk of death (HR, 0.46 [95% CI, 0.23–0.91]; *P* = 0.025) remained in metformin users compared to non-metformin users.

**Table 2:** Baseline characteristics of 511 diabetic patients with chronic obstructive pulmonary disease, stratified by metformin use

Characteristic	Total	Metformin	Non-metformin	P value
	*N* = 511	*N* = 282	*N* = 229	
Age, years	73.0 ± 9.4	72.1 ± 9.3	74.0 ± 9.3	0.025
≥ 65	407 (80)	211 (75)	196 (86)	0.003
Male gender	419 (82)	232 (82)	187 (82)	0.858
BMI (*N* = 482; 266; 216)	24.6 ± 4.3	24.8 ± 4.4	24.4 ± 4.1	0.251
< 18.5	31 (6.4)	17 (6.4)	14 (6.5)	0.712
18.5–24	198 (41)	105 (40)	93 (43)	
> 24	253 (53)	144 (54)	109 (51)	
GOLD stage				
1	139 (27)	88 (31)	51 (22)	0.110
2	242 (47)	127 (45)	115 (50)	
3	115 (23)	61 (22)	54 (24)	
4	15 (2.9)	6 (2.1)	9 (3.9)	
Comorbidity				
Hypertension	309 (61)	165 (59)	144 (63)	0.315
Cerebrovascular disease	56 (11)	29 (10)	27 (12)	0.588
Heart failure	72 (14)	22 (7.8)	50 (22)	< 0.001
Coronary artery disease	164 (32)	84 (30)	80 (35)	0.215
Malignancy	92 (18)	42 (15)	50 (22)	0.042
Hospitalization, No.^a^	0.04 ± 0.22	0.03 ± 0.17	0.06 ± 0.27	0.113
≥ 1	20 (3.9)	8 (2.8)	12 (5.2)	0.164
HbA1c, % (*N* = 494; 275; 219)	7.1 ± 1.1	7.1 ± 1.1	7.1 ± 1.2	0.972
Antidiabetic class, No.				
< 2	401 (79)	192 (68)	209 (91)	< 0.001
≥ 2	110 (22)	90 (32)	20 (8.7)	

*BMI* body mass index, *GOLD* Global Initiative for Chronic Obstructive Lung Disease, *HbA1c* haemoglobin A1c

^a^Within 1 year after the index date

**Fig. 4:**
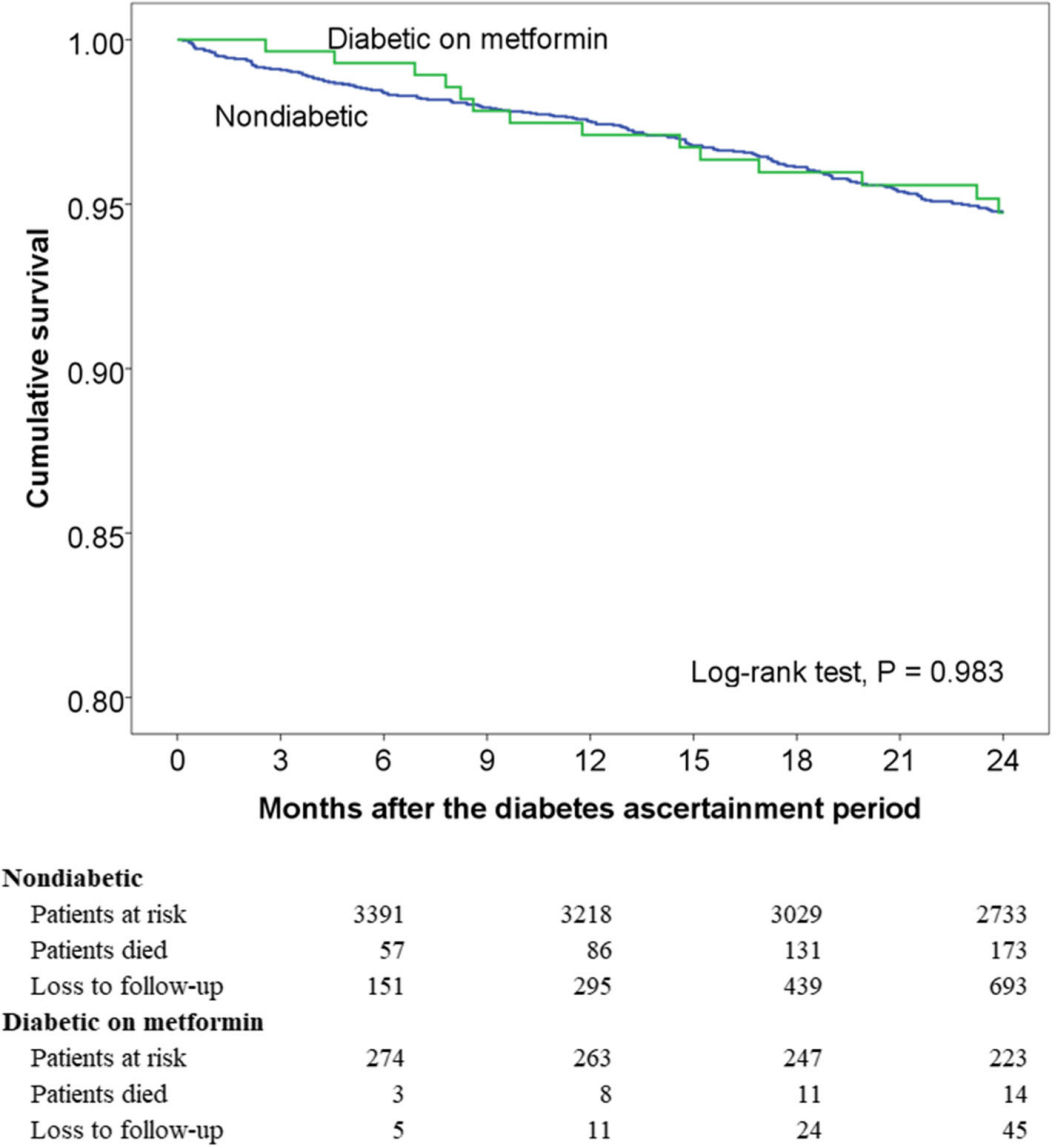
Unadjusted Kaplan-Meier estimates of time to mortality in chronic obstructive pulmonary disease, comparing diabetic patients on metformin to nondiabetic patients (*N* = 3881). The mean follow-up period of the patients was 21.4 months, with 83,098 person-months at risk. Patients with chronic kidney disease stage 3b or higher were excluded due to concerns for confounding

Subsequently, the 282 DM patients on metformin during the 2-year follow-up period were compared to the remaining 3599 COPD patients without DM. Patient mortality was not significantly different between the two groups in the log-rank test of the Kaplan-Meier survival curves (*P* = 0.983; Fig. 4). In the Cox regression model controlling for age, gender, GOLD stage, comorbidities, and prior COPD hospitalization (global test *P* = 0.254), DM patients being placed on metformin had no difference in hazards of death compared with COPD patients without DM during the follow-up period (HR, 0.98 [95% CI, 0.56–1.69]; *P* = 0.927; Additional file 5).

## Discussion

The most prominent findings of this study are that (a) COPD patients with coexistent DM had a significantly higher hazard of death during a 2-year follow-up period than those without; (b) metformin use was associated with a favourable outcome in terms of all-cause mortality among patients with COPD and DM; (c) metformin appeared to reverse the adverse prognostic effect of DM on survival in COPD patients. In short, our data suggest that metformin, if not contraindicated, may be the drug of choice for the treatment of DM in the presence of COPD.

In a study including patients with COPD exacerbations and DM, metformin use was associated with a survival benefit although potential confounders were not properly accounted for in the analysis [28]. A recent work using a nationwide claims database by Yen et al. further proved that a lower risk of mortality was observed for metformin users despite acknowledging inherent drawbacks of insurance database, such as lack of information on BMI, pulmonary function tests, and renal function in patients with DM and either stable or exacerbated COPD [29]. Another clinical study also showed that metformin use in patients with coexistent COPD and DM is associated with lower healthcare utilization, defined as emergency room visits and hospitalization [30]. Moreover, a prospective observational study demonstrated that metformin improves the symptomatology and functional status among COPD patients with DM or impaired glucose tolerance [20]. In line with the aforementioned findings, the present large-scale study provided further evidence that metformin users had a better survival outcome than non-metformin users after adjusting for relevant observable characteristics in patients with COPD and DM. The plausible biological explanations for the beneficial effects of metformin in the coexistence of COPD and DM are not clearly understood but may involve its pleiotropic features. Via a variety of signalling pathways, metformin is reportedly able to inhibit anti-transforming growth factor-β-induced lung fibrosis, to attenuate oxidative stress and pro-inflammatory cytokine responses to reduce airway inflammation and remodelling and to enhance or maintain the expression of anti-inflammatory cytokines [31–33]. In addition, skeletal muscle dysfunction is a common phenomenon in patients with COPD or DM [34, 35]. Metformin, through mediating activation of adenosine monophosphate-activated protein kinase, improves mitochondrial biogenesis and function to increase glucose disposal and muscle glycogen concentrations and consequently enhances muscle function [36, 37]. Taken together, despite the background mechanisms remain to be elucidated, clinical and experimental evidence indicates that metformin, an inexpensive, off-patent, and widely used antidiabetic drug, is a reasonable and viable choice for COPD patients with DM.

Evidence indicates that DM as a comorbidity is associated with disease progression and worse prognosis of COPD patients because of its adverse effects on lung anatomy and physiology, inflammatory responses, and antimicrobial defenses [9]. In consistency with these findings, the present study found that COPD patients with DM had a significantly higher hazard of 2-year mortality compared with those without. Furthermore, recent evidence supports that COPD is an important risk factor for the development of DM [38–40]. Thus, it has been advocated that targeted surveillance and intervention measures for DM are needed in the clinical care of COPD patients although this strategy is not recommended in the current practice guidelines of COPD [2, 10, 39]. Notably and interestingly, our study added to the existing knowledge by showing that COPD patients with DM and receiving metformin had a similar hazard of death to COPD patients without DM. The finding suggests that metformin use in patients with coexistent COPD and DM may even reverse the negative prognostic impact of DM on COPD outcomes.

This study has a couple of limitations. First, the relationships between chronic systemic diseases are complicated. Whether beneficial outcomes of metformin are due to its favourable glucose homeostasis action, protective effects on the cardiovascular system, or direct influence on the natural course of COPD remains uncertain and is beyond the scope of this study. However, we observed a survival advantage associated with metformin use even after controlling for important clinical confounders. Second, the study findings from a single site, thereby eliminating variance in clinical practice and availability of medical resources, may not be generalizable to other institutions or settings. Yet, as a large study to date demonstrating the survival benefit of metformin among COPD patients, we hope that our report will draw attention to this specific patient population and encourage more studies to investigate this issue across a variety of research settings.

## Conclusions

In summary, this study shows that DM as a comorbidity is associated with an increased risk of mortality in patients with COPD after adjustment for age, gender, GOLD stage, comorbidities and prior COPD hospitalization. Our analysis further indicates that metformin use was associated with lower hazards of death among patients with coexistent COPD and DM, and may mitigate the negative prognostic effect of DM on COPD, suggesting the potential role for this agent in the management of DM in the setting of COPD; however, prospective randomized studies are needed to validate and confirm our results.

## Additional file

Additional file 1: A table showing the prescriptions of antidiabetic drugs other than metformin among patients with diabetes mellitus. (DOCX 16 kb)

Additional file 2: A table showing adjusted hazard ratios based on the Cox proportional hazards model of 2-year mortality in chronic obstructive pulmonary disease. (DOCX 17 kb)

Additional file 3: A table showing the diagnostic criteria for diabetes mellitus in the study population. (DOCX 16 kb)

Additional file 4: A table showing adjusted hazard ratios based on the Cox proportional hazards model of 2-year mortality among diabetic patients in chronic obstructive pulmonary disease. (DOCX 17 kb)

Additional file 5: A table showing adjusted hazard ratios based on the Cox proportional hazards model of 2-year mortality in chronic obstructive pulmonary disease, comparing diabetic patients on metformin to nondiabetic patients. (DOCX 16 kb)

## Abbreviations

BMI: Body mass index
CI: Confidence interval
CKD: Chronic kidney disease
COPD: chronic obstructive pulmonary disease
DM: Diabetes mellitus
FVC: Forced vital capacity
GOLD: Global Initiative for Chronic Obstructive Lung Disease
HbA1c: Hemoglobin A1c
HR: Hazard ratio
NTUH-iMD: Integrated Medical Database, National Taiwan University Hospital
SD: Standard deviation

## References

[1] Lopez AD, Shibuya K, Rao C, Mathers CD, Hansell AL, Held LS, Schmid V, Buist S (2006). Chronic obstructive pulmonary disease: current burden and future projections. Eur Respir J.

[2] Vogelmeier CF, Criner GJ, Martinez FJ, Anzueto A, Barnes PJ, Bourbeau J, Celli BR, Chen R, Decramer M, Fabbri LM, Frith P, Halpin DM, Lopez Varela MV, Nishimura M, Roche N, Rodriguez-Roisin R, Sin DD, Singh D, Stockley R, Vestbo J, Wedzicha JA, Agusti A (2017). Global strategy for the diagnosis, management and prevention of chronic obstructive lung disease 2017 report: GOLD executive summary. Respirology.

[3] Agusti AG (2005). Systemic effects of chronic obstructive pulmonary disease. Proc Am Thorac Soc.

[4] Rabe KF, Hurd S, Anzueto A, Barnes PJ, Buist SA, Calverley P, Fukuchi Y, Jenkins C, Rodriguez-Roisin R, van Weel C, Zielinski J (2007). Global initiative for chronic obstructive lung D. Global strategy for the diagnosis, management, and prevention of chronic obstructive pulmonary disease: GOLD executive summary. Am J Respir Crit Care Med.

[5] Miller J, Edwards LD, Agusti A, Bakke P, Calverley PM, Celli B, Coxson HO, Crim C, Lomas DA, Miller BE, Rennard S, Silverman EK, Tal-Singer R, Vestbo J, Wouters E, Yates JC, Macnee W (2013). Evaluation of CLtIPSEI. Comorbidity, systemic inflammation and outcomes in the ECLIPSE cohort. Respir Med.

[6] Mannino DM, Watt G, Hole D, Gillis C, Hart C, McConnachie A, Davey Smith G, Upton M, Hawthorne V, Sin DD, Man SF, Van Eeden S, Mapel DW, Vestbo J (2006). The natural history of chronic obstructive pulmonary disease. Eur Respir J.

[7] Wild S, Roglic G, Green A, Sicree R, King H (2004). Global prevalence of diabetes: estimates for the year 2000 and projections for 2030. Diabetes Care.

[8] Parappil A, Depczynski B, Collett P, Marks GB (2010). Effect of comorbid diabetes on length of stay and risk of death in patients admitted with acute exacerbations of COPD. Respirology.

[9] Glaser S, Kruger S, Merkel M, Bramlage P, Herth FJ (2015). Chronic obstructive pulmonary disease and diabetes mellitus: a systematic review of the literature. Respiration.

[10] Ho TW, Huang CT, Ruan SY, Tsai YJ, Lai F, Yu CJ (2017). Diabetes mellitus in patients with chronic obstructive pulmonary disease-the impact on mortality. PLoS One.

[11] Singanayagam A, Schembri S, Chalmers JD (2013). Predictors of mortality in hospitalized adults with acute exacerbation of chronic obstructive pulmonary disease. Ann Am Thorac Soc.

[12] McGhan R, Radcliff T, Fish R, Sutherland ER, Welsh C, Make B (2007). Predictors of rehospitalization and death after a severe exacerbation of COPD. Chest.

[13] American Diabetes A (2013). Standards of medical care in diabetes--2013. Diabetes Care.

[14] Formoso G, De Filippis EA, Michetti N, Di Fulvio P, Pandolfi A, Bucciarelli T, Ciabattoni G, Nicolucci A, Davi G, Consoli A (2008). Decreased in vivo oxidative stress and decreased platelet activation following metformin treatment in newly diagnosed type 2 diabetic subjects. Diabetes Metab Res Rev.

[15] Haffner S, Temprosa M, Crandall J, Fowler S, Goldberg R, Horton E, Marcovina S, Mather K, Orchard T, Ratner R, Barrett-Connor E (2005). Diabetes prevention program research G. Intensive lifestyle intervention or metformin on inflammation and coagulation in participants with impaired glucose tolerance. Diabetes.

[16] Garnett JP, Baker EH, Naik S, Lindsay JA, Knight GM, Gill S, Tregoning JS, Baines DL (2013). Metformin reduces airway glucose permeability and hyperglycaemia-induced Staphylococcus aureus load independently of effects on blood glucose. Thorax.

[17] Baker EH, Janaway CH, Philips BJ, Brennan AL, Baines DL, Wood DM, Jones PW (2006). Hyperglycaemia is associated with poor outcomes in patients admitted to hospital with acute exacerbations of chronic obstructive pulmonary disease. Thorax.

[18] Kupeli E, Ulubay G, Ulasli SS, Sahin T, Erayman Z, Gursoy A (2010). Metabolic syndrome is associated with increased risk of acute exacerbation of COPD: a preliminary study. Endocrine.

[19] Kim HJ, Lee JY, Jung HS, Kim DK, Lee SM, Yim JJ, Yang SC, Yoo CG, Chung HS, Kim YW, Han SK, Shim YS, Lee CH (2010). The impact of insulin sensitisers on lung function in patients with chronic obstructive pulmonary disease and diabetes. Int J Tuberc Lung Dis.

[20] Sexton P, Metcalf P, Kolbe J (2014). Respiratory effects of insulin sensitisation with metformin: a prospective observational study. COPD.

[21] Vestbo J, Hurd SS, Agusti AG, Jones PW, Vogelmeier C, Anzueto A, Barnes PJ, Fabbri LM, Martinez FJ, Nishimura M, Stockley RA, Sin DD, Rodriguez-Roisin R (2013). Global strategy for the diagnosis, management, and prevention of chronic obstructive pulmonary disease: GOLD executive summary. Am J Respir Crit Care Med.

[22] Inzucchi SE, Lipska KJ, Mayo H, Bailey CJ, McGuire DK (2014). Metformin in patients with type 2 diabetes and kidney disease: a systematic review. JAMA.

[23] Almagro P, Salvado M, Garcia-Vidal C, Rodriguez-Carballeira M, Delgado M, Barreiro B, Heredia JL, Soriano JB (2010). Recent improvement in long-term survival after a COPD hospitalisation. Thorax.

[24] American Diabetes A (2016). 7. Approaches to glycemic treatment. Diabetes Care.

[25] Levey AS, de Jong PE, Coresh J, El Nahas M, Astor BC, Matsushita K, Gansevoort RT, Kasiske BL, Eckardt KU (2011). The definition, classification, and prognosis of chronic kidney disease: a KDIGO controversies conference report. Kidney Int.

[26] Weinberg CR (1993). Toward a clearer definition of confounding. Am J Epidemiol.

[27] Cheng SL, Chan MC, Wang CC, Lin CH, Wang HC, Hsu JY, Hang LW, Chang CJ, Perng DW, Yu CJ (2015). COPD in Taiwan: a National Epidemiology Survey. Int J Chron Obstruct Pulmon Dis.

[28] Hitchings AW, Archer JR, Srivastava SA, Baker EH (2015). Safety of metformin in patients with chronic obstructive pulmonary disease and type 2 diabetes mellitus. COPD.

[29] Yen FS, Chen W, Wei JC, Hsu CC, Hwu CM (2018). Effects of metformin use on total mortality in patients with type 2 diabetes and chronic obstructive pulmonary disease: a matched-subject design. PLoS One.

[30] Bishwakarma R, Zhang W, Lin YL, Kuo YF, Cardenas VJ, Sharma G (2018). Metformin use and health care utilization in patients with coexisting chronic obstructive pulmonary disease and diabetes mellitus. Int J Chron Obstruct Pulmon Dis.

[31] Cameron AR, Morrison VL, Levin D, Mohan M, Forteath C, Beall C, McNeilly AD, Balfour DJ, Savinko T, Wong AK, Viollet B, Sakamoto K, Fagerholm SC, Foretz M, Lang CC, Rena G (2016). Anti-inflammatory effects of metformin irrespective of Diabetes status. Circ Res.

[32] Park CS, Bang BR, Kwon HS, Moon KA, Kim TB, Lee KY, Moon HB, Cho YS (2012). Metformin reduces airway inflammation and remodeling via activation of AMP-activated protein kinase. Biochem Pharmacol.

[33] Sato N, Takasaka N, Yoshida M, Tsubouchi K, Minagawa S, Araya J, Saito N, Fujita Y, Kurita Y, Kobayashi K, Ito S, Hara H, Kadota T, Yanagisawa H, Hashimoto M, Utsumi H, Wakui H, Kojima J, Numata T, Kaneko Y, Odaka M, Morikawa T, Nakayama K, Kohrogi H, Kuwano K (2016). Metformin attenuates lung fibrosis development via NOX4 suppression. Respir Res.

[34] Jones SE, Maddocks M, Kon SS, Canavan JL, Nolan CM, Clark AL, Polkey MI, Man WD (2015). Sarcopenia in COPD: prevalence, clinical correlates and response to pulmonary rehabilitation. Thorax.

[35] Sreekumar R, Nair KS (2007). Skeletal muscle mitochondrial dysfunction & diabetes. Indian J Med Res.

[36] Musi N, Hirshman MF, Nygren J, Svanfeldt M, Bavenholm P, Rooyackers O, Zhou G, Williamson JM, Ljunqvist O, Efendic S, Moller DE, Thorell A, Goodyear LJ (2002). Metformin increases AMP-activated protein kinase activity in skeletal muscle of subjects with type 2 diabetes. Diabetes.

[37] Frayn KN, Adnitt PI (1972). Effects of metformin on glucose uptake by isolated diaphragm from normal and diabetic rats. Biochem Pharmacol.

[38] Cazzola M, Bettoncelli G, Sessa E, Cricelli C, Biscione G (2010). Prevalence of comorbidities in patients with chronic obstructive pulmonary disease. Respiration.

[39] Lin CS, Liu CC, Yeh CC, Chang YC, Chung CL, Lane HL, Shih CC, Chen TL, Liao CC (2017). Diabetes risks and outcomes in chronic obstructive pulmonary disease patients: two nationwide population-based retrospective cohort studies. PLoS One.

[40] Meteran H, Backer V, Kyvik KO, Skytthe A, Thomsen SF (2015). Comorbidity between chronic obstructive pulmonary disease and type 2 diabetes: a nation-wide cohort twin study. Respir Med.

