# Supplementary material for: Metformin use mitigates the adverse prognostic effect of diabetes mellitus in chronic obstructive pulmonary disease
Source: Respir Res. 2019 Apr 5;20:69. doi: 10.1186/s12931-019-1035-9 (PMC6451256; doi:10.1186/s12931-019-1035-9)
Supplement: Supplementary file 1 — A table showing the prescriptions of antidiabetic drugs other than metformin among patients with diabetes mellitus. (DOCX 16 kb) [file 12931_2019_1035_MOESM1_ESM.docx]

Additional file 1. Prescriptions of antidiabetic drugs other than metformin among patients with diabetes mellitus

|  | Total | Metformin | Non-metformin |
| --- | --- | --- | --- |
| Antidiabetic drugs | N = 511 | N = 282 | N = 229 |
| Metformin | 282 (55) | 282 (100) | 0 (0) |
| Sulfonylurea | 186 (36) | 71 (25) | 115 (50) |
| Dipeptidyl peptidase-4 inhibitor | 53 (10) | 20 (7.1) | 33 (14) |
| α-glucosidase inhibitor | 45 (8.8) | 14 (5.0) | 31 (14) |
| Insulin | 33 (6.5) | 10 (3.5) | 23 (10) |
| Thiazolidinedione | 30 (5.9) | 16 (5.7) | 14 (6.1) |
| Meglitinide | 29 (5.7) | 11 (3.9) | 18 (7.9) |
| None | 25 (4.9) | 0 (0) | 25 (11) |
