# Supplementary material for: Metformin use mitigates the adverse prognostic effect of diabetes mellitus in chronic obstructive pulmonary disease
Source: Respir Res. 2019 Apr 5;20:69. doi: 10.1186/s12931-019-1035-9 (PMC6451256; doi:10.1186/s12931-019-1035-9)
Supplement: Supplementary file 2 — A table showing adjusted hazard ratios based on the Cox proportional hazards model of 2-year mortality in chronic obstructive pulmonary disease. (DOCX 17 kb) [file 12931_2019_1035_MOESM2_ESM.docx]

Additional file 2. Adjusted hazard ratios based on the Cox proportional hazards model of 2-year mortality in chronic obstructive pulmonary disease (N = 4231)

| Characteristic | Adjusted HR^*^ | 95% CI | P value |
| --- | --- | --- | --- |
| Diabetes mellitus | 1.62 | 1.15-2.28 | 0.006 |
| Age, ≥65 years | 1.75 | 1.11-2.76 | 0.015 |
| Male gender | 0.89 | 0.59-1.35 | 0.584 |
| GOLD stage |  |  |  |
| 2 vs. 1 | 1.00 | 0.70-1.43 | 0.989 |
| 3 vs. 1 | 1.11 | 0.72-1.71 | 0.637 |
| 4 vs. 1 | 1.54 | 0.75-3.17 | 0.239 |
| Comorbidity |  |  |  |
| Hypertension | 1.12 | 0.82-1.52 | 0.491 |
| Cerebrovascular disease | 0.85 | 0.52-1.38 | 0.503 |
| Heart failure | 1.50 | 1.02-2.21 | 0.040 |
| Coronary artery disease | 0.85 | 0.59-1.22 | 0.382 |
| Malignancy | 2.60 | 1.89-3.57 | <0.001 |
| Chronic kidney disease | 1.88 | 1.27-2.79 | 0.002 |
| Hospitalization, No. ≥1^‡^ | 3.16 | 1.95-5.11 | <0.001 |

CI, confidence interval; GOLD, Global Initiative for Chronic Obstructive Lung Disease; HR, hazard ratio.

^*^ Adjusted for all variables included in the table.

^‡^ Within 1 year after the index date.
