# Supplementary material for: Metformin use mitigates the adverse prognostic effect of diabetes mellitus in chronic obstructive pulmonary disease
Source: Respir Res. 2019 Apr 5;20:69. doi: 10.1186/s12931-019-1035-9 (PMC6451256; doi:10.1186/s12931-019-1035-9)
Supplement: Supplementary file 3 — A table showing the diagnostic criteria for diabetes mellitus in the study population. (DOCX 16 kb) [file 12931_2019_1035_MOESM3_ESM.docx]

Additional file 3. Diagnostic criteria for diabetes mellitus in the study population

| Criteria | N (%) of patients |
| --- | --- |
| ICD codes plus a prescription of oral antidiabetics or insulin | 527 (95) |
| ICD codes plus |  |
| (a)+(b)+(c)^*^ | 3 (0.5) |
| (a)+(c) | 13 (2.3) |
| (b)+(c) | 13 (2.3) |

ICD, International Classification of Diseases.

^*^ (a) Random blood glucose ≥200 mg/dl; (b) Haemoglobin A1c ≥6.5%; (c) Fasting blood glucose ≥126 mg/dl.
