# Supplementary material for: Metformin use mitigates the adverse prognostic effect of diabetes mellitus in chronic obstructive pulmonary disease
Source: Respir Res. 2019 Apr 5;20:69. doi: 10.1186/s12931-019-1035-9 (PMC6451256; doi:10.1186/s12931-019-1035-9)
Supplement: Supplementary file 4 — A table showing adjusted hazard ratios based on the Cox proportional hazards model of 2-year mortality among diabetic patients in chronic obstructive pulmonary disease. (DOCX 17 kb) [file 12931_2019_1035_MOESM4_ESM.docx]

Additional file 4. Adjusted hazard ratios based on the Cox proportional hazards model of 2-year mortality among diabetic patients in chronic obstructive pulmonary disease (N = 511)

| Characteristic | Adjusted HR^*^ | 95% CI | P value |  |
| --- | --- | --- | --- | --- |
| Metformin use | 0.46 | 0.23-0.92 | 0.028 |  |
| Age, ≥65 years | 3.54 | 1.07-11.70 | 0.038 |  |
| Male gender | 0.98 | 0.42-2.26 | 0.956 |  |
| GOLD stage |  |  |  |  |
| 2 vs. 1 | 0.74 | 0.33-1.65 | 0.459 |  |
| 3 vs. 1 | 1.29 | 0.55-3.05 | 0.561 |  |
| 4 vs. 1 | 0.49 | 0.08-3.05 | 0.447 |  |
| Comorbidity |  |  |  |  |
| Hypertension | 1.10 | 0.57-2.15 | 0.772 |  |
| Cerebrovascular disease | 1.23 | 0.50-3.04 | 0.653 |  |
| Heart failure | 1.37 | 0.61-3.06 | 0.442 |  |
| Coronary artery disease | 0.82 | 0.41-1.67 | 0.588 |  |
| Malignancy | 2.27 | 1.14-4.51 | 0.019 |  |
| Hospitalization, No. ≥1^‡^ | 7.91 | 2.74-22.92 | <0.001 |  |
| Antidiabetic class, ≥2 | 0.91 | 0.39-2.12 | 0.825 |  |

CI, confidence interval; GOLD, Global Initiative for Chronic Obstructive Lung Disease; HR, hazard ratio.

^*^ Adjusted for all variables included in the table.

^‡^ Within 1 year after the index date.
