# Supplementary material for: Metformin use mitigates the adverse prognostic effect of diabetes mellitus in chronic obstructive pulmonary disease
Source: Respir Res. 2019 Apr 5;20:69. doi: 10.1186/s12931-019-1035-9 (PMC6451256; doi:10.1186/s12931-019-1035-9)
Supplement: Supplementary file 5 — A table showing adjusted hazard ratios based on the Cox proportional hazards model of 2-year mortality in chronic obstructive pulmonary disease, comparing diabetic patients on metformin to nondiabetic patients. (DOCX 16 kb) [file 12931_2019_1035_MOESM5_ESM.docx]

Additional file 5. Adjusted hazard ratios based on the Cox proportional hazards model of 2-year mortality in chronic obstructive pulmonary disease, comparing diabetic patients on metformin to nondiabetic patients (N = 3881)

| Characteristic | Adjusted HR^*^ | 95% CI | P value |
| --- | --- | --- | --- |
| Metformin use | 0.98 | 0.56-1.69 | 0.927 |
| Age, ≥65 years | 1.51 | 1.02-2.23 | 0.039 |
| Male gender | 0.80 | 0.52-1.21 | 0.287 |
| GOLD stage |  |  |  |
| 2 vs. 1 | 0.99 | 0.70-1.41 | 0.971 |
| 3 vs. 1 | 1.19 | 0.79-1.79 | 0.415 |
| 4 vs. 1 | 1.94 | 0.99-3.81 | 0.055 |
| Comorbidity |  |  |  |
| Hypertension | 0.95 | 0.70-1.30 | 0.750 |
| Cerebrovascular disease | 1.35 | 0.84-2.16 | 0.214 |
| Heart failure | 2.12 | 1.41-3.19 | <0.001 |
| Coronary artery disease | 0.87 | 0.59-1.29 | 0.500 |
| Malignancy | 3.41 | 2.51-4.64 | <0.001 |
| Hospitalization, No. ≥1^‡^ | 2.37 | 1.42-3.99 | 0.001 |

CI, confidence interval; GOLD, Global Initiative for Chronic Obstructive Lung Disease; HR, hazard ratio.

^*^ Adjusted for all variables included in the table.

^‡^ Within 1 year after the index date.
